# Supplementary material for: Analysis of PPARγ Signaling Activity in Psoriasis
Source: Int J Mol Sci. 2021 Aug 10;22(16):8603. doi: 10.3390/ijms22168603 (PMC8395241; doi:10.3390/ijms22168603)
Supplement: Supplementary file 1 [file ijms-22-08603-s001.zip › Supplemental materials_Analysis of PPARg signaling activity in psoriasis/Pathway models/Models images and html files/PPARG inhibits phenotypes assotiated with psoriasis/135291.html]

toxicity


# Disease toxicity

|  |  |
| --- | --- |
| URN | urn:agi-disease:toxicity%20(disease) |
| Total Entities | 27 |
| Connectivity | 15508 |
| Name | toxicity |

---

|  |  |
| --- | --- |
| ChildConcepts | rare disorder due to toxic effects |
|  | poisoning |
|  | organ toxicity |
|  | drug toxicity |
|  | phytotoxicity |
|  | developmental toxicity |
|  | acute toxicity |
|  | chronic toxicity |
|  | grade 2 toxicity |
|  | grade 3 toxicity |
|  | grade 4 toxicity |
|  | grade 5 toxicity |
|  | hypertoxicity |
|  | infusional toxicity |
|  | late toxicity |
|  | long-term toxicity |
|  | grade 1 toxicity |
|  | overall toxicity |
|  | regimen-related toxicity |
|  | short-term toxicity |
|  | treatment-related toxicity |
|  | endocrine toxicity |
|  | central nervous system toxicity |
|  | retinal toxicity |
|  | gastric toxicity |
|  | glucotoxicity |
|  | phototoxicity |

---

|  |  |
| --- | --- |
| Pathway | PPARG inhibits phenotypes assotiated with psoriasis |

---

|  |  |
| --- | --- |
| MedScan ID | 9035749 |

---

|  |  |
| --- | --- |
| Alias | toxic reactions |
|  | toxic reaction |
|  | toxic event |
|  | toxigenicities |
|  | toxicity |
|  | toxic events |
|  | toxic actions |
|  | toxicities |
|  | toxic action |
|  | toxic effects |
|  | toxigenicity |
|  | toxic effect |

---
